# Identification of Ubiquitin Genes and Their Expression Patterns in *Pinus massoniana* Under Infection Stress from the Pinewood Nematode

**DOI:** 10.3390/plants15071106

**Published:** 2026-04-03

**Authors:** Qingyang Chen, Haiyu Zhou, Shan Hu, Zhichun Zhou, Bin Liu, Kai Gao, Kongshu Ji, Qinghua Liu

**Affiliations:** 1 State Key Laboratory of Tree Genetics and Breeding, Nanjing Forestry University, Nanjing 210037, China; 13345012802@163.com; 2 Research Institute of Subtropical Forestry, Chinese Academy of Forestry, Hangzhou 311400, China; 15029702710@163.com (H.Z.); lkyhushan@163.com (S.H.); zczhou_risf@163.com (Z.Z.); lb_binbin@163.com (B.L.); gaokai@caf.ac.cn (K.G.); 3 National Key Laboratory of Forest Genetics and Tree Breeding, Chinese Academy of Forestry, Beijing 100091, China

**Keywords:** *Pinus massoniana*, ubiquitin-like protein, *UBQ* gene family, RPL40, pine wilt disease

## Abstract

Ubiquitins (Ubs) play a crucial role in plant–pathogen interactions, particularly the *RPL40* family, which is essential for protein synthesis. While *Pinus massoniana* is highly susceptible to pine wilt disease (PWD) caused by *Bursaphelenchus xylophilus*, the defense mechanisms mediated by RPL40s remain poorly understood. Here, we performed a genome-wide identification of the ubiquitin and ubiquitin-like gene family (*PmUBQs*) in *P. massoniana*. We identified 30 *PmUBQ* genes unevenly distributed across 11 chromosomes, which were classified into six subfamilies based on phylogenetic analysis. An analysis of promoter regions indicated that the *PmUBQ* genes were enriched with cis-acting elements associated with stress responses, hormone regulation, and development. Specifically, two group II members, *PmRPL40-1* and *PmRPL40-2*, located on chromosomes 12 and 11, respectively, were identified and exhibited distinct responses to *B. xylophilus* infection in resistant and susceptible *P. massoniana*. *PmRPL40-1* was significantly highly expressed in the 15 days post-inoculation, while *PmRPL40-2* was downregulated on day 1 and then upregulated. Moreover, both genes showed peak divergence at 15 days post-inoculation; the expression levels of *PmRPL40-1* and *PmRPL40-2* in resistant *P. massoniana* were approximately 1.8- and 3.7-fold higher, respectively, than in susceptible *P. massoniana*. These patterns suggest that *PmRPL40s* might be involved in the rapid activation of defense responses and late-stage cell repair. Notably, transient overexpression of *PmRPL40-1* in *P. massoniana* led to a significant 1.6-fold increase in the jasmonic acid (JA) content (*p* < 0.0001). These findings reveal the key *PmUBQ* genes and suggest that *PmRPL40s* contribute to PWD resistance potentially through the modulation of JA signaling, offering potential targets for molecular breeding in *P. massoniana*.

## 1. Introduction

*Pinus massoniana* Lamb., an evergreen coniferous tree belonging to the genus *Pinus* of the family *Pinaceae* [1], serves as a principal afforestation species in southern China and is extensively utilized in land greening, pulp and paper manufacturing, and the construction and panel industries. The resin and pollen of *P. massoniana* are important raw materials for the chemical industry and health products [2]. However, this species is highly susceptible to pine wilt disease (PWD) caused by the pinewood nematode *Bursaphelenchus xylophilus*. This disease is characterized by high mortality rates, rapid transmission, and difficulties in control, which severely hinder the development of the *P. massoniana* industry. Therefore, in-depth research into the response mechanisms of *P. massoniana* to nematode stress is essential for providing new insights into genetic improvements for disease resistance.

Ubiquitin (Ub) is a small protein composed of 76 amino acids; it is highly conserved and ubiquitous in all eukaryotic cells [3]. Based on domain architecture, Ub-related proteins can be categorized into four types: monoubiquitins, polyubiquitins, ubiquitin–ribosomal fusion proteins, and ubiquitin–ubiquitin-like fusion proteins [4,5]. In *Arabidopsis thalian*, 77 members of the ubiquitin gene family have been identified, each encoding proteins containing at least one ubiquitin domain. Among these, 16 are classified as polyubiquitin genes, 4 as ubiquitin–ribosomal fusion genes, and 3 as RUB (related to ubiquitin) ubiquitin-like genes [6,7].

The ubiquitination pathway, also referred to as the Ub pathway or ubiquitin/26S proteasome pathway, involves a series of specific enzymes that identify cellular proteins, select target proteins, and mediate their specific modification. Ubiquitin genes encode the ubiquitin protein, and their expression levels and sequence characteristics are closely related to the efficiency of ubiquitination. Through the ubiquitin–proteasome system (UPS), formed by the conjugation of ubiquitin to E3 ubiquitin ligases, ubiquitination is broadly involved in plant growth, development, and stress responses. Ubiquitination plays crucial roles in protein localization, metabolism, function, regulation, and degradation, and it is involved in the regulation of nearly all biological processes, including the cell cycle, proliferation, apoptosis, differentiation, migration, gene expression, transcriptional regulation, signal transduction, damage repair, and inflammatory immunity. Monoubiquitin primarily functions in transcriptional regulation, DNA damage repair, and DNA replication; polyubiquitin is mainly involved in protein degradation; and ubiquitin–ribosomal fusion proteins are principally responsible for protein synthesis [8,9,10,11,12,13,14,15]. High temperatures upregulate *UbL40* mRNA levels. A previous study found that, in the *tms5* mutant, loss of ZS1 function led to excessive accumulation of *UbL40* mRNA, resulting in reduced pollen production and male sterility. In contrast, in wild-type plants, excess UbL4* mRNA was cleaved by ZS1, maintaining normal fertility [14]. Transgenic plants overexpressing ribosomal proteins (RPs) exhibit enhanced resistance to multiple pathogens. A previous study found that overexpression of *AtRPL10A* did not confer complete resistance to the non-host pathogen *Pseudomonas syringae* pv. but prolonged survival compared to wild-type plants. Mutants or RNAi lines of *AtRPL10A*, *B*, and *C* showed reduced resistance to non-host *P. syringae* pv [15]. In tobacco (*Nicotiana tabacum*), *NtUBC* and *NtUBQ* enhanced salt tolerance by boosting 26S proteasome activity, thereby reducing Na^+^ accumulation, reactive oxygen species (ROS), and ubiquitinated/salt-denatured proteins [16]. Overexpression of wheat TaRub strengthened disease resistance in *Arabidopsis* [4]. Knockout of *SlPUB22* in tomato (*Solanum lycopersicum*) increased susceptibility to *Botrytis cinerea* and attenuated jasmonic acid (JA)-induced primary root growth inhibition and anthocyanin accumulation. These JA responses were largely rescued by mutation of *JAZ4* (Jasmonate ZIM-domain 4), indicating that PUB22-JAZ4 broadly participates in multiple JA-responsive processes [17]. Ribosomal proteins play significant roles in growth, development, and responses to biotic and abiotic stresses; however, the function of *RPL40s* in plant biotic stress resistance remains inadequately studied. Elucidating their functions is critical for identifying molecular targets for disease resistance.

In this study, we employed bioinformatics approaches, integrating Pfam domain models for ubiquitin and ubiquitin-like proteins, the Ubl_ubiquitin domain, and *P. massoniana* genome data to identify the *PmUBQ* gene family for the first time. We characterized the candidate *PmUBQ* family members in terms of protein physicochemical properties, phylogenetic relationships, conserved motifs, and chromosomal locations. Furthermore, we analyzed the expression patterns of key *PmUBQ* genes in highly resistant and susceptible *P. massoniana* lines at different stages following pinewood nematode infection. The function of *PmRPL40-1* was validated via transient transformation. The aim of this study was to elucidate the key roles of *PmUBQ* gene family members in the response to pine wilt disease, providing a foundation for unraveling the disease resistance mechanisms in *P. massoniana*.

## 2. Results

### 2.1. Identification and Physicochemical Property Analysis of the PmUBQs Gene Family Members

The *UBQ* gene family members of *A. thaliana*, *O. sativa*, and *P. tremuloides* were compared with the genomic data of *P. massoniana*. A total of 231 *PmUBQ* candidate sequences were obtained. Sequences without Ubl_ubiquitin domains, sequences with incomplete domains, and identical sequences were excluded. Finally, 30 gene sequences were obtained. The family members were named based on the number of Ubl_ubiquitin domains in the protein. The molecular weight of the encoded proteins of the *PmUBQ* gene family members ranged from 8650.03 to 85,313.91 Da, the number of encoded amino acid residues ranged from 77 to 762, the isoelectric point ranged from 5.25 to 10.23, the instability index ranged from 14.15 to 51.47, the fatty acid index ranged from 74.36 to 105.14, the hydrophilicity ranged from −0.878 to −0.075, and the hydrophobic index was less than 0, indicating that all PmUBQ proteins are hydrophilic. Subcellular localization prediction showed that all 30 *PmUBQ* family members were located in the nucleus and cytoplasm (Table 1).

### 2.2. Analysis of Conserved Motifs, Conserved Domains, and Gene Structure of the PmUBQ Gene Family

**Figure 1:**
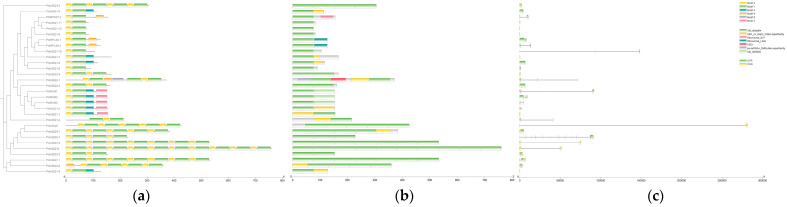
Gene structure, conserved motifs, and conserved domains of the *PmUBQ* gene family in *P. massoniana* (**a**): Conserved motif of protein encoded by *PmUBQ* gene. (**b**): Conserved domain diagram. (**c**): Gene structure map.

The conserved motifs of the *PmUBQ* family proteins were characterized, and 10 motifs (motif 1–motif 10) were identified. All 30 members of the *PmUBQ* family had motif 1 and 2 (Figure 1a). The conserved domains were predicted using NCBI-CDD, and all 30 members contained the conserved domain CD01803, indicating that they all belong to the ubiquitin family (Figure 1b).

Gene structure analysis showed that the exon number of the *PmUBQ* gene was 1–15, and most family members had 2–3 exons. Among them, the exon number of *PmUBQ3-1* was the largest, reaching up to 15. Among the 5 single-ubiquitin genes in the *UBQ* family, only one exon was present in each gene, and 3 genes (*PmUBQ1-4*, *PmUBQ1-10*, *PmUBQ1-11*) lacked the untranslated regions (UTRs) (Figure 1c).

### 2.3. Phylogenetic Analysis of the PmUBQ Gene Family in P. massoniana

**Figure 2:**
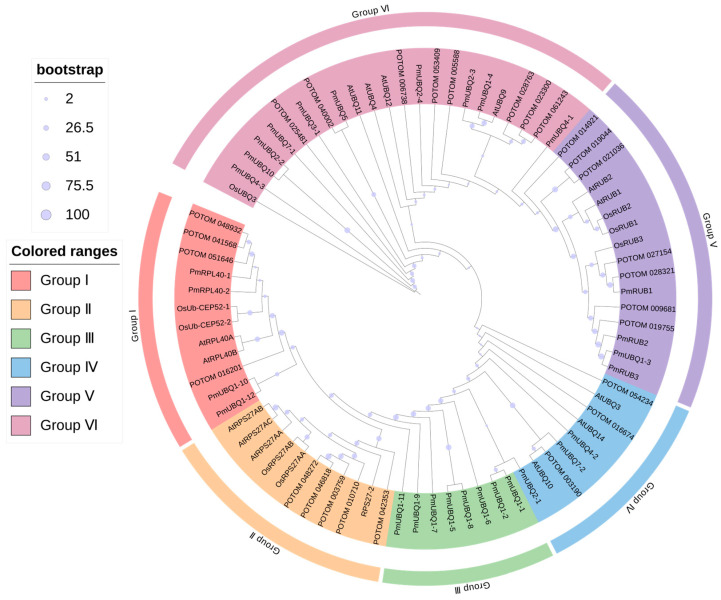
Phylogenetic tree of *PmUBQ* gene family. Pm: *P. massoniana*; POTOM: *P. tomentosa*; At: *A. thaliana*; Os: *O. sativa.*

The UBQ protein sequences of four plant species—*P. massoniana*, *O. sativa*, *A. thaliana*, and *P. tomentosa*—totaling 89 sequences, were aligned. A phylogenetic tree was constructed usingIQTREE, and the *PmUBQ* family members were classified based on phylogenetic relationships and protein domains. The results (Figure 2) showed that the 89 UBQ genes could be clustered into six groups (Groups I–VI). Group I primarily contained UBQ + RPL40 domains, including four *P. massoniana* genes, two *A. thaliana* genes, two *O. sativa* genes, and four *P. tomentosa* genes. Group II mainly contained UBQ + RPS27 domains, comprising one *P. massoniana* genes. Group III mainly consisted of monoubiquitin genes, including eight genes from *Pinus massoniana*. Group IV included three *P. massoniana* polyubiquitin genes. Group V primarily contained UBQ + Nedd8 domains, consisting of four *P. massoniana* genes. Group VI mainly consisted of polyubiquitin protein chains formed by the covalent linkage of the same type of UBQ monomers, containing ten *P. massoniana* genes. As shown in the figure, *PmUBQ* genes were more closely related to *P. tomentosa* than to *A. thaliana* and *O. sativa*, indicating higher conservation of UBQ genes among woody plants than among herbaceous plants. The classification into Groups I, II, III, IV, V, and VI was primarily based on sequence homology and phylogenetic relationships.

### 2.4. Chromosomal Localization of the PmUBQ Gene Family in P. massoniana

**Figure 3:**
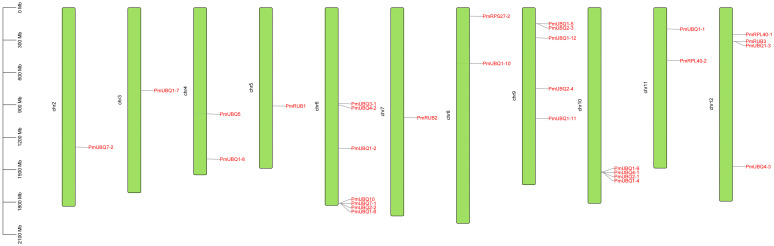
Chromosomal distribution of *PmUBQ* gene family in *P. massoniana.*

To determine the distribution of *UBQ* gene family members in the *P. massoniana* genome, data on chromosome lengths and gene positions were extracted. The chromosomal locations of *PmUBQ* genes were plotted using TBtools software. The results (Figure 3) indicate that the 30 *PmUBQ* genes are distributed across 11 chromosomes in an uneven manner. A majority of these genes are located at the terminal ends of the chromosomes, with varying quantities: Chr2 to Chr12 contain 1, 1, 2, 1, 7, 1, 2, 5, 4, 2, and 4 genes, respectively. Notably, the highest number of genes is found on Chr6. The genes on Chr6 and Chr10 tended to cluster closely, with only a few dispersed across the chromosomes, suggesting large-scale duplication of *PmUBQ* genes in the *P. massoniana* genome.

### 2.5. Analysis of Cis-Acting Elements in the Promoter of PmUBQ Gene Family

**Figure 4:**
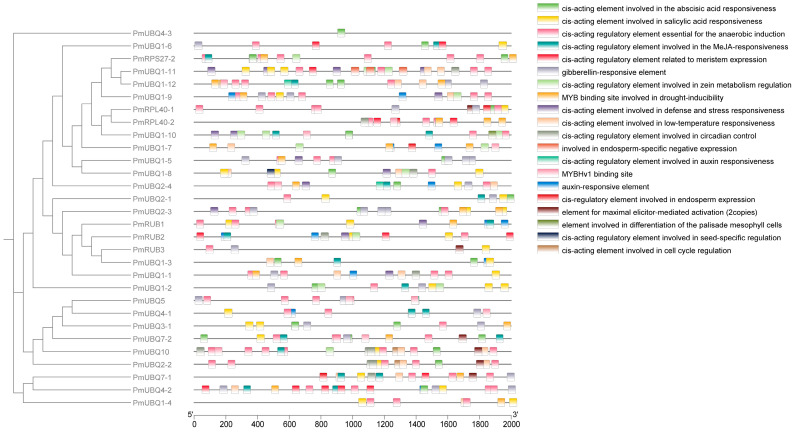
Sites of cis-acing elements of *PmUBQ* gene family in *P. massoniana*.

The promoters of *P. massoniana* UBQ contained a diverse array of cis-acting elements, mainly including light-responsive elements, phytohormone-responsive elements (abscisic acid, methyl jasmonate, auxin, and gibberellin), transcription factor-related-responsive elements, growth and development-responsive elements (seed development, endosperm expression, cell differentiation, and cell cycle regulation), and stress-responsive elements (drought, low temperature, defense and stress, and wounding). Among the promoters of all gene family members, A total of 392 responsive elements were identified, comprising 167 phytohormone-responsive elements, 40 growth and development-responsive elements, 147 transcription factor-related-responsive elements, and 36 stress-responsive elements (Figure 4). Genes such as *PmRUB2*, *PmUBQ2-3*, and *PmUBQ1-11* contain cis-acting elements that are involved in defense and stress responses. Additionally, genes such as *PmUBQ1-2* and *PmUBQ1-12* were found to have MeJA-responsive elements, while *PmUBQ10* and *PmUBQ1-11* harbor SA-responsive elements. The presence of these elements may provide insights into the role of the *PmUBQ* family in the disease resistance of *P. massoniana*.

### 2.6. Analysis of Expression Patterns of PmUBQs in Highly Resistant and Susceptible P. massoniana in Response to B. xylophilus Infection

**Figure 5:**
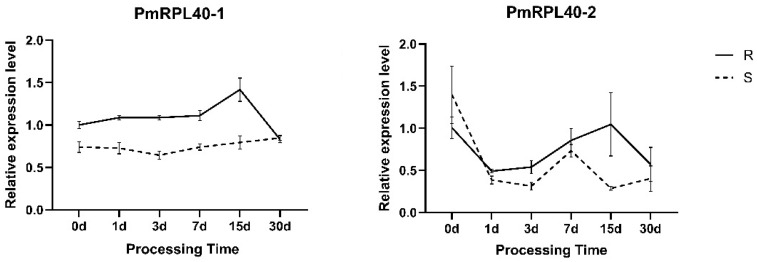
Expression levels of different genes under *Bursaphelenchus xylophilus* treatment. The expression of *PmRPs* genes was validated using qRT-PCR. Relative expression was normalized to the disease-resistant plant inoculated for 0 days (R0). The data are presented as the mean ± standard error of three biological replicates. Asterisks indicate significant differences in expression between disease-resistant and -susceptible plants (*p* < 0.05).

Ribosomes play an integral part in plant growth, development, and defense responses. The function of ubiquitin–ribosomal fusion proteins in plant immunity has not yet been fully elucidated. To investigate their response characteristics in *P. massoniana* under *B. xylophilus* stress, two *P. massoniana* genes from Group II in the phylogenetic tree, namely, *PmRPL40-1* and *PmRPL40-2*, were selected as the research objects, and their expression levels were verified using quantitative real-time PCR (qRT-PCR) (Figure 5). *PmRPL40-1* showed a weak response in the early stages of infection; its expression level was upregulated in the highly resistant families at 15 days post-inoculation (dpi) and then downregulated to the same level as that in the susceptible families at 30 dpi, while the expression level of *PmRPL40-1* in the susceptible families was significantly lower than that in the highly resistant families within the first 15 dpi. The expression of *PmRPL40-2* exhibited an overall decreasing–increasing–decreasing trend: it was higher in the susceptible families than in the highly resistant families at 0 dpi, and it was significantly lower in the susceptible families than in the highly resistant families at all other time points. These results indicate that *PmUBQs* play an important role in the immune response of *P. massoniana* to *B. xylophilus* infection.

### 2.7. Transient Transformation and Functional Analysis of Pinus massoniana Seedlings Mediated by PmRPL40-1

**Figure 6:**
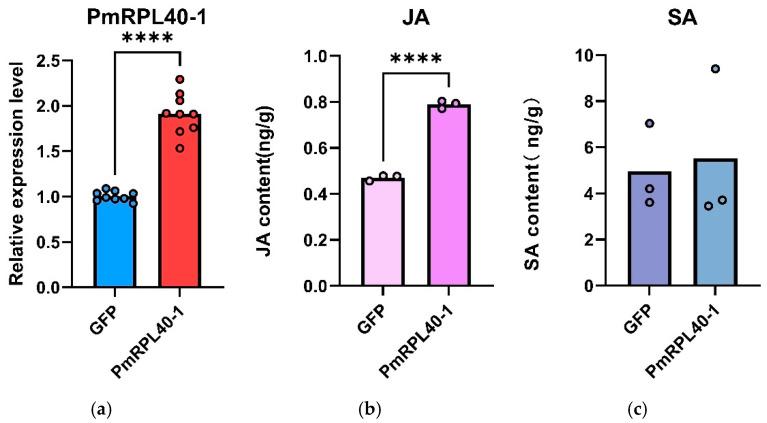
Changes in hormone contents of *P. massoniana* seedlings after transient overexpression of *PmRPL40-1*. (**a**): Detection of gene expression in transiently transformed *P. massoniana* seedlings. (**b**): Changes in jasmonic acid (JA) content. (**c**): Changes in salicylic acid (SA) content. The data are presented as the mean ± standard error of three biological replicates. Asterisks indicate significant differences between transgenic plants and control plants (**** *p* < 0.0001; independent samples *t*-test).

To further investigate the role of the PmRPL40-1 gene in activating the defense response of *P. massoniana* against *B. xylophilus*, an overexpression vector of this gene was constructed and used for the transient transformation of *P. massoniana* calli. The qRT-PCR results (Figure 6a) showed that the relative expression level of *PmRPL40-1* in calli transiently transformed with pCAMBIA1300-PmRPL40-1-EGFP was significantly higher than that in the control group (calli transiently transformed with the empty vector), with a 1.9-fold increase (*p* < 0.0001), indicating the successful transient transformation of *P. massoniana* calli. Endogenous phytohormones are important components of the plant immune system. We further examined the changes in the jasmonic acid (JA) and salicylic acid (SA) contents in the transformed calli, and the results (Figure 6b,c) showed that the former was upregulated by 1.6-fold (*p* < 0.0001) after transient overexpression of *PmRPL40-1*, while the latter showed no significant change.

**Figure 7:**
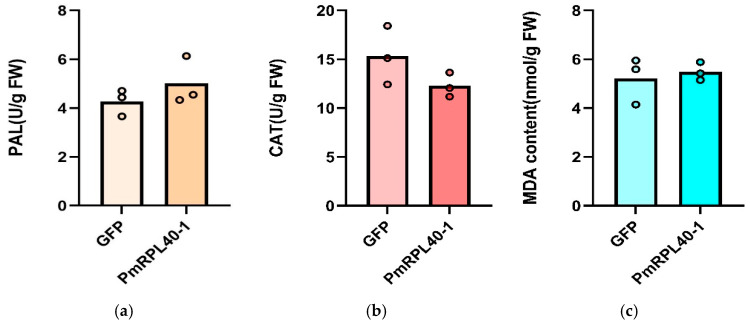

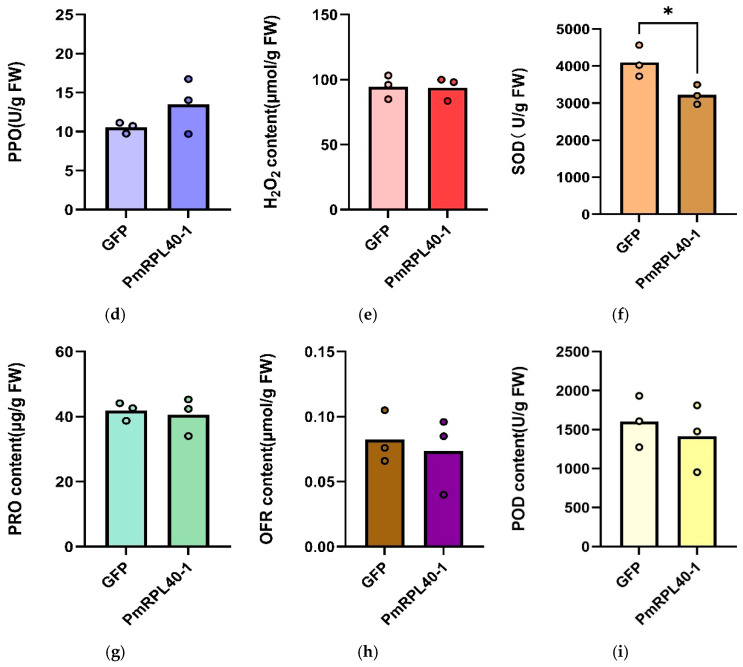
Physiological indicators of *P. massoniana* seedling after transient overexpression of *PmRPL40-1.* (**a**): Changes in phenylalanine ammonia-lyase (PAL); (**b**): Catalase (CAT); (**c**): Malondialdehyde (MDA); (**d**): Polyphenol oxidase (PPO); (**e**): Hydrogen peroxide (H_2_O_2_); (**f**): Superoxide dismutase (SOD); (**g**): Proline (PRO); (**h**): Superoxide anion radical (OFR); (**i**): Peroxidase (POD). The data are presented as the mean ± standard error of three biological replicates. Asterisks indicate significant differences between transgenic plants and control plants (* *p* < 0.05; independent samples *t*-test).

Superoxide dismutase (SOD), polyphenol oxidase (PPO), and their metabolism-related enzymes are key physiological and biochemical indicators in plants during pathogen infection, and they are closely associated with disease resistance. The results of this study (Figure 7) showed that overexpression of *PmRPL40-1* reduced SOD activity and slightly increased activity of PPO. No significant changes were observed in the activities of other enzymes.

## 3. Discussion

Ubiquitin (UBQ) acts as a key player in plant disease resistance against the invasion of bacterial, fungal, viral and other pathogens by regulating critical processes including the activation of immune signaling pathways, pathogen recognition, and synthesis of defense substances. To date, 77 ubiquitin family genes have been identified in *A. thaliana* [7], whereas research on Pinaceae plants, especially *P. massoniana*, remains extremely limited. Therefore, based on the published genome data of *P. massoniana*, this study conducted the first identification of members of *PmUBQs* gene family. Combined with the analysis of the types and numbers of conserved amino acid domains, a total of 30 UBQ proteins were screened out from *P. massoniana*, and all members contained a complete Ubl_ubiquitin conserved domain.

An analysis of the physicochemical properties and gene structures of *P. massoniana PmUBQ* genes revealed certain differences in the physicochemical properties of proteins encoded by the different members of this gene family, which might be related to the complex recombination or selection events experienced by the ubiquitin family during the evolutionary process [18]. The results of a conserved motif analysis showed that the composition and arrangement patterns were consistent within the same subfamily but varied among different subfamilies, which was consistent with the functional differences in different ubiquitin subfamilies [19]. Subcellular localization prediction indicated that all proteins encoded by the family members were localized in the nucleus and cytoplasm, which was consistent with the main sites where ubiquitination occurs [20]. In this study, 12 out of the 30 members of the *P. massoniana PmUBQ* gene family were found to be concentrated on Chr6 and Chr9, accounting for 40% of the total, indicating the presence of large-scale UBQ gene duplication in the *P. massoniana* genome. Five monoubiquitin genes, including *PmUBQ1-4*, *PmUBQ1-10*, and *PmUBQ1-11,* had no introns. Previous studies have demonstrated that introns are usually absent in the coding regions of monoubiquitin genes [21,22], and intronless genes generally exhibit faster expression [23]. This structural feature may facilitate the early and rapid expression of monoubiquitin genes to activate defense responses.

The promoter regions of the PmUBQ gene family contained a variety of important response elements, including defense- and stress-related elements, as well as phytohormone-responsive elements associated with disease resistance such as MeJA and SA. The presence of these elements suggests that *PmUBQ* genes have the potential to regulate plant disease resistance. As a signaling molecule, JA participates in regulating plant disease resistance responses and alleviating the effects of abiotic stress [24]. The ubiquitin ligase CUL3^BPME^3 can regulate the stability of MYC2, MYC3, and MYC4 proteins, thereby modulating the JA signaling pathway [25]. In transgenic citrus plants overexpressing Csubiquitin and CsRub2, the JA content is significantly downregulated, and JA signaling can negatively regulate citrus canker resistance by antagonizing SA-mediated disease resistance [26].

Although a large body of evidence indicates that PmUBQs act as defense proteins in plant–pathogen interactions, their biological functions remain unclear. Based on the transcriptome analysis of highly resistant and susceptible *P. massoniana* at different time points after inoculation, combined with qRT-PCR results, it was found that the *PmUBQ* gene family exhibited distinct expression trends in response to *B. xylophilus* infection. *PmRPL40-1* and *PmRPL40-2* reached the highest expression level at 15 days post-inoculation, suggesting that *PmRPL40s* may be involved in late-stage cell repair [27]. The delayed expression response in susceptible families led to cell death and subsequent wilting. Transgenic plants overexpressing ribosomal proteins (RPs) exhibit enhanced resistance to many pathogens. Overexpression of *AtRPL10A* failed to confer complete resistance to the non-host pathogen *P. syringae* pv, but the survival time was longer than that of wild-type plants. Mutants or RNAi lines of *AtRPL10A*, *B*, and *C* showed reduced resistance to the non-host pathogen *P. syringae* pv [15]. The high expression of the ubiquitin–ribosomal genes *PmRPL40-1* and *PmRPL40-2* in highly resistant families may enhance the resistance of *P. massoniana* to pine wilt disease. Plant resistance to pathogens often relies on the precise temporal and spatial regulation of immune signals, and the timely activation of the ubiquitination system is critical for the efficient initiation of defense responses. In tomato, the histone monoubiquitination genes SlHUB1 and SlHUB2 can mediate the monoubiquitination of histone H2B, and the silencing of SlHUB1 or SlHUB2 genes increases the susceptibility of tomato to *Botrytis cinerea* [28]. Rice SPL11 can mediate the ubiquitination and degradation of OsSDS2 (SPL11 cell-death suppressor 2), while OsSDS2, in turn, can phosphorylate SPL11. Meanwhile, OsSDS2 phosphorylates OsRbohB and OsRLCK118 to regulate ROS production, thereby positively modulating rice PCD and disease resistance immune responses [29]. *PmRPS27-1* exhibited high expression levels within the first 15 days of inoculation, which may enhance the efficiency of ubiquitination modification and accelerate the degradation of negative regulatory factors or the functional activation of defense proteins, thus timely inhibiting the nematode infection process. In contrast, the initial decrease in expression levels in susceptible families delayed signal activation, leading to the failure to effectively suppress the pathogenic pressure of *B. xylophilus* and subsequent plant death.

Plant disease resistance and growth and development are generally considered energy-consuming processes. In plants, the enhancement of disease resistance responses can affect growth and development, a phenomenon known as the trade-off between disease resistance and growth and development. In the absence of pathogen infection, disease resistance genes usually maintain only low expression levels and are in an inactive state; however, their overexpression or activation has adverse effects on growth and development. For example, in *A. thaliana* SNC1 gain-of-function mutants, the increased expression and activity of disease resistance genes lead to dwarfism, leaf crinkling, and reduced seed setting rates; continuous overexpression of the *Capsicum annuum* disease resistance gene Bs3 or the *Hordeum vulgare* disease resistance gene Rph3 can induce plant programmed cell death [30,31,32]. The expression level of *PmRPL40-2* in susceptible families was significantly higher than that in highly resistant families at 0 days post-inoculation. The high basal expression of this group of genes in susceptible families may lead to the overactivation of the ubiquitination system, triggering the excessive degradation of non-disease resistance proteins, thereby affecting the growth and development of the plants; the drastic fluctuations after infection may further disrupt the balance between growth and immunity, leading to the loss of control of defense responses. In contrast, the stable expression of this group of genes in highly resistant families may ensure the precise regulation of defense-related proteins (such as PR proteins) by maintaining a moderate intensity of ubiquitination modification without affecting the normal growth of the plants, thus achieving sustained resistance to *B. xylophilus*.

Jasmonic acid is an important plant signaling molecule that plays a key role in plant disease resistance responses. Exogenous JAs can significantly upregulate the expression of PR4, PR5, and PEROX genes in wheat, enhancing its resistance to *Fusarium graminearum* [33]. After pest and disease infestation in plants, the JA signaling pathway is activated, JA-mediated transcriptional reprogramming is initiated and amplified in a cascade manner, the transduction of downstream signals is triggered to produce defense responses, the expression of resistance genes is induced, and chemical substances involved in defense are further synthesized [17,34]. In plants, ROS play a crucial role in abiotic and biotic stress responses, as well as in signal transduction [35]. Not only can ROS directly damage pathogens and induce plant programmed cell death to limit pathogen spread, but they can also act as signaling molecules to transmit signals and synergize with other defense substances [36,37]. SOD is a core key enzyme in the plant antioxidant defense system that can scavenge excessive reactive oxygen species. In this study, overexpression of *PmRPL40-1* increased the JA content and slightly downregulated SOD activity in *P. massoniana* seedlings. These results suggest that this gene may be related to JA accumulation and reduced basal antioxidant capacity in *P. massoniana*, indicating its potential involvement in the JA signaling pathway.

This study systematically analyzed the structure of *PmUBQ* family members and their response characteristics to *B. xylophilus* infection. Among them, *PmRPL40-1* and *PmRPL40-2* play important roles in the resistance of highly resistant *P. massoniana* to *B. xylophilus* infection, and they can serve as key candidate genes to provide gene targets for elucidating the disease resistance functions of *PmUBQs* and developing new disease-resistant *P. massoniana* germplasm.

This study also has certain limitations. Due to time constraints, multiple testing corrections were not applied in the validation analyses, nor were additional family members functionally verified. These shortcomings will be thoroughly addressed in future research.

## 4. Materials and Methods

### 4.1. Materials

The materials were collected from 5-year-old *P. massoniana* clonal trial forests in Linhai Nursery, Linhai City, Zhejiang Province. Three plants each of a highly resistant clonal line (Huang13-1) and a susceptible clonal line (He9-5) were selected, and their current-year tender branches were inoculated with *Bursaphelenchus xylophilus*. The nematodes used were a mixture of the highly pathogenic “Guangde 3B” and a nematode population isolated from diseased *P. massoniana* trees in the wild. Each tender branch was inoculated with 10,000 nematodes/200 µL. At 0 d, 1 d, 7 d, 15 d, and 30 d after inoculation, three biological replicates of the highly resistant and susceptible *P. massoniana* clonal lines were collected from the inoculation sites, immediately placed in liquid nitrogen, and used for subsequent experiments.

### 4.2. Methods

#### 4.2.1. Identification, Physicochemical Properties, and Subcellular Localization Prediction Analysis of PmUBQ Family Members in *P. massoniana*

The *P. massoniana* genome data used in this study were obtained from a publicly available dataset published by Guangxi Academy of Forestry (PRJNA1240911). The hidden Markov model of the ubiquitin and ubiquitin-like conserved domain (Pfam ID: PF00240) was downloaded from the InterPro 108.0 website (https://www.ebi.ac.uk/interpro/entry/pfam/PF00240/logo/ (accessed on 3 January 2026)), and the protein sequences of the *UBQ* family in *Arabidopsis thaliana*, *Oryza sativa*, and *Populus tomentosa* were obtained from the same website (https://www.ebi.ac.uk/interpro/entry/pfam/PF00240/protein/UniProt/#table (accessed on 3 January 2026)). Subsequently, HMM Search (E-value ≤ 1 × 10^−10^) was performed using TBtools v2.441 to preliminarily screen candidate gene family members. Using the obtained UBQ protein sequences of *A. thaliana*, *O. sativa*, and *P. tomentosa* as queries, BLASTP analysis was conducted against the *P. massoniana* genome database with Tbtools-II v2.411, applying a strict threshold (E-value ≤ 1 × 10^−10^). The batch CD search of the NCBI database (https://www.ncbi.nlm.nih.gov/Structure/bwrpsb/bwrpsb.cgi (accessed on 3 January 2026)) was further used to analyze conserved domains. Sequences lacking the UBQ conserved domain or containing incomplete conserved domains were removed. Gene sequences with the same number of bases were aligned using SnapGene v6.0.2 to eliminate redundant sequences. CD-HIT V4.8.1 was employed to eliminate redundant sequences, utilizing the following parameters: a sequence identity threshold of 90%, an alignment coverage of at least 80% for both longer and shorter sequences, and the accurate clustering mode. The physicochemical properties of the *PmUBQ* family members, including sequence length, protein isoelectric point, and relative molecular mass, were analyzed using the ExPAS website (https://web.expasy.org/protparam/ (accessed on 4 January 2026)), and the nuclear localization signal was predicted using NLS Mapper (http://nls-mapper.iab.keio.ac.jp/cgi-bin/NLS_Mapper_form.cgi (accessed on 4 January 2026)). The subcellular localization of the *PmUBQ* family members was predicted using the online software WoLF PSORT (https://wolfpsort.hgc.jp/ (accessed on 4 January 2026)).

#### 4.2.2. Motif, Gene Structure, and Conserved Domain Analysis of the PmUBQ Gene Family in *P. massoniana*

The extracted *PmUBQ* gene family members were submitted to the MEME website, with the number of motifs set to 10, to identify conserved motifs. The conserved structures were determined using NCBI-CDD (https://www.ncbi.nlm.nih.gov/Structure/bwrpsb/bwrpsb.cgi (accessed on 5 January 2026)), and the number of exons and introns was predicted using TBtools. In this study, we first performed multiple sequence alignments of multispecies sequences using MAFFT v7.505 with default parameters. Based on the alignment results, we employed IQ-TREE2 v2.0.7 to construct a maximum-likelihood phylogenetic tree. During the tree construction process, ModelFinder was utilized to automatically select the optimal evolutionary model based on the Bayesian Information Criterion (BIC), ultimately adopting the VT+R3 model for analysis. The reliability of the phylogenetic tree was assessed using 1000 ultrafast bootstrap replicates (-B 1000). Visualization was performed using TBtools software.

#### 4.2.3. Phylogenetic Tree Construction and Phylogenetic Analysis of the *PmUBQ* Gene Family in *Pinus massoniana*

A total of 14 *Arabidopsis thaliana* UBQ protein sequences, 27 *Populus tomentosa* carrière UBQ protein sequences, and 8 *Oryza sativa* UBQ protein sequences were downloaded from the NCBI database TAIR (https://www.arabidopsis.org/ (accessed on 6 January 2026)). In this study, we first performed multiple sequence alignments of multispecies sequences using MAFFT v7.505 with default parameters. Based on the alignment results, we employed IQ-TREE2 v2.0.7 to construct a maximum-likelihood phylogenetic tree. During the tree construction process, ModelFinder was utilized to automatically select the optimal evolutionary model based on the Bayesian Information Criterion (BIC), ultimately adopting the VT+R3 model for analysis. The reliability of the phylogenetic tree was assessed using 1000 ultrafast bootstrap replicates (-B 1000). The constructed phylogenetic tree was visualized and optimized using the online tool Interactive Tree Of Life (iTOL, https://itol.embl.de/ (accessed on 6 January 2026)).

#### 4.2.4. Chromosome Localization of the PmUBQ Gene Family in *P. massoniana*

The genomic positions of the *PmUBQ* gene family members were extracted from the gff file of the *P. massoniana* genome, and a chromosome gene distribution map was constructed using TBtools software.

#### 4.2.5. Prediction of Cis-Regulatory Elements in the PmUBQ Gene Family of *P. massoniana*

The 2 kb upstream region of the start codon of each *PmUBQ* gene was extracted from the gff file of the annotated *P. massoniana* genome and considered the promoter sequence. The cis-regulatory elements within the promoters were predicted through the PlantCARE database, and the results were visualized using TBtools.

#### 4.2.6. Analysis of the Gene Expression Pattern of PmUBQs in *P. massoniana* Under *B. xylophilus* Stress

The pinewood nematode was inoculated, and the needles of highly resistant and susceptible *P. massoniana* were extracted. RNA was extracted using an RN38 EASY Spin Plus Plant RNA Rapid Extraction Kit (Edle, Beijing, China), according to the manufacturer’s instructions, and it was reverse-transcribed using PrimeScript™ RT Master Mix (Perfect Real Time) (TaKaRa, Beijing, China). Fluorescent quantitative primers were designed in the non-conserved domain region of the gene using Primer3plus (https://www.primer3plus.com/ (accessed on 13 January 2026)) (Table 2), and the diluted cDNA was used as the template. TB Green^®^ Premix Ex Taq™ II (Tli RNaseH Plus) (TaKaRa, Beijing, China) was used according to the manufacturer’s instructions. The reaction was performed using the ABI PRISM 7300 Real-Time PCR System (Foster City, CA, USA), with *P. massoniana* EF2 used as the internal reference gene, and 3 biological and 3 technical replicates were carried out. Relative expression levels were calculated using the 2^−ΔΔCT^ method. Data were analyzed and plotted using GraphPad Prism software v10.1.2. Two-way ANOVA was performed, followed by multiple comparisons and normality was tested using the Shapiro–Wilk test.

#### 4.2.7. Transient Transformation of *P. massoniana* Seedlings with PmRPL40-1

The *PmRPL40-1* gene was cloned and ligated into the pCAMBIA1300-35S-EGFP expression vector via homologous recombination. Both the empty vector and the constructed recombinant plasmid pCAMBIA1300-PmRPL40-1-35S-EGFP were transformed into Agrobacterium tumefaciens strain GV3101, which was cultured in LB medium at 28 °C with shaking until the OD_600_ value reached 0.6–0.8. Following centrifugation, the bacterial cells were resuspended in a resuspension solution (Murashige and Skoog medium + 200 μmol/L acetosyringone) to an OD_600_ of 0.1. Masson pine block calli were immersed in the infection solution and subjected to vacuum infiltration for 10 min. After filtering out the bacterial suspension with a sterile cell strainer, the explants were blotted dry with filter paper and placed on solid co-culture medium. After 48 h of co-cultivation in the dark, the calli were harvested, immediately frozen in liquid nitrogen, and stored at −80 °C. The contents of SA, JA, MDA, PRO, OFR, and H_2_O_2_, as well as SOD, CAT, POD, PPO, and PAL, were determined using corresponding assay kits from Kwinbon Biotechnology Co., Ltd. (Nanjing, China). Each sample weighed 2 g, and three biological replicates were performed. Data analysis and graphing were conducted using GraphPad Prism software. Data were analyzed using independent samples *t*-test, and normality was tested using the Shapiro–Wilk test.

## 5. Conclusions

This study identified a total of 30 *PmUBQ* gene family members, which were unevenly distributed across the 11 chromosomes of *P. massoniana*. A number of these family members showed tandem duplications on chromosomes 6 and 10. The promoter regions of the *PmUBQ* genes were rich in plant hormone response elements and stress-responsive cis-acting elements. Furthermore, under *B. xylophilus* (pinewood nematode) stress, *PmUBQs* in both highly resistant, and susceptible *P. massoniana* exhibited either co-expression or specific expression patterns, indicating functional diversification within the *PmUBQ* family. Specifically, *PmRPL40-1* expression was significantly higher in the highly resistant families than in the susceptible ones at 15 days post-inoculation with *B. xylophilus*, whereas *PmRPL40-2* expression initially decreased and then increased. These findings suggest that *PmUBQ* family members underwent functional divergence during evolution, potentially initiating the immune response in *P. massonian* by regulating different biological processes. Transient overexpression of *PmRPL40-1* can upregulate JA content and downregulate SOD activity. This research provides insights for further elucidating the interaction mechanisms between pine trees and the pinewood nematode.

## Figures and Tables

**Table 1:** Physical and chemical properties of *PmUBQ* gene family.

Gene	Number of Amino Acid	Molecular Weight	Theoretical pI	Instability Index	Aliphatic Index	Grand Average of Hydropathicity
*PmRPL40-1*	128	14,673.28	9.94	32.82	89.92	−0.638
*PmRPL40-2*	128	14,673.28	9.94	32.82	89.92	−0.638
*PmRPS27-2*	156	17,877.84	9.77	36.05	74.36	−0.846
*PmRUB1*	153	17,192.72	5.97	50.61	100	−0.512
*PmRUB2*	153	17,154.67	6.17	37.06	100.59	−0.509
*PmRUB3*	153	17,170.85	7.82	24.11	101.83	−0.378
*PmUBQ10*	762	85,313.91	6.85	29.23	101.56	−0.442
*PmUBQ1-* *6*	114	12,658.55	5.88	14.15	101.75	−0.354
*PmUBQ1-* *7*	168	18,830.42	8.71	35.99	84.7	−0.506
*PmUBQ1-* *8*	92	10,433.02	6.57	25.95	98.48	−0.375
*PmUBQ1-* *9*	84	9610.06	9.4	33.26	96.31	−0.56
*PmUBQ1-1* *0*	106	12,050.17	10.23	51.47	74.53	−0.878
*PmUBQ1-1* *1*	84	9535.97	7.93	48.49	98.57	−0.37
*PmUBQ1-1* *2*	77	8650.03	6.71	32.35	105.06	−0.288
*PmUBQ1-* *1*	156	17,518.07	5.25	46.76	98.08	−0.413
*PmUBQ1-* *2*	216	23,655.26	6.42	51.04	105.14	−0.075
*PmUBQ1-* *3*	154	17,132.7	6.91	28.22	94.22	−0.399
*PmUBQ1-* *4*	129	14,447.68	6.74	29.44	98.99	−0.369
*PmUBQ1-* *5*	118	13,120.11	7.97	22.33	104.07	−0.341
*PmUBQ2-1*	372	42,290.63	6.22	39.8	93.52	−0.448
*PmUBQ2-* *2*	154	17,259.79	6.14	28.98	102.53	−0.432
*PmUBQ2-* *3*	162	18,449.07	6.31	30.09	95.68	−0.537
*PmUBQ2-* *4*	168	19,139.98	6.31	26.59	98.63	−0.448
*PmUBQ3-1*	229	25,685.49	6.86	29.17	100.87	−0.431
*PmUBQ4-1*	384	43,085.59	8.28	28.38	100.49	−0.388
*PmUBQ4-* *2*	361	40,308.46	8.45	27.79	103.66	−0.362
*PmUBQ4-* *3*	307	34,403.49	6.48	30.1	101.6	−0.438
*PmUBQ5*	426	47,998.14	8.86	34.34	99.98	−0.371
*PmUBQ7-1*	534	59,813.61	6.71	29.22	100.94	−0.445
*PmUBQ7-* *2*	533	59,695.53	7.09	28.52	101.13	−0.444

Note: All gene family members were predicted to be localized in the nucleus and cytoplasm.

**Table 2:** Primers used for RT-qPCR.

Gene	Primer Sequence
*PmRPL40-1-F*	GCACTCTGGCGGATTACAAC
*PmRPL40-1-R*	GCCCACACTTCTTTTTCCTGC
*PmRPL40-2-F*	GAGTCTACGCTGCACCTTGT
*PmRPL40-2-R*	CCGCACTTCTTTTTCCTGCA
*PmEF2-F*	CTGCGATGTCCCTCATGTTA
*PmEF2-R*	AACAAGGTCTTTCCCCTCGT

## Data Availability

The original contributions presented in this study are included in the article. Further inquiries can be directed to the corresponding author.
